# Sheltering Role of Well-Decayed Conifer Logs for Forest Floor Fungi in Long-Term Polluted Boreal Forests

**DOI:** 10.3389/fmicb.2021.729244

**Published:** 2021-10-06

**Authors:** Vladimir S. Mikryukov, Olesya V. Dulya, Igor E. Bergman, Georgiy A. Lihodeevskiy, Anzhelika D. Loginova, Leho Tedersoo

**Affiliations:** ^1^Institute of Plant and Animal Ecology, Ural Branch of the Russian Academy of Sciences, Yekaterinburg, Russia; ^2^Department of Botany, Chair of Mycology, Institute of Ecology and Earth Sciences, Tartu, Estonia; ^3^Laboratory of Molecular Biology, Ural State Agricultural University, Yekaterinburg, Russia; ^4^Mycology and Microbiology Center, University of Tartu, Tartu, Estonia

**Keywords:** coarse woody debris, diversity maintenance, environmental pollution, fungi, heavy metal, metabarcoding, soil

## Abstract

Coarse woody debris (CWD) provides food and shelter to a large proportion of forest biota and is considered vital for biodiversity during periods of harsh weather. However, its importance in long-term stressed ecosystems remains largely unknown. In this work, we explored the contribution of CWD to fungal diversity along the gradient of boreal forest degradation caused by 77 years of heavy industrial emissions. We analyzed the diversity and composition of fungi in 270 samples of well-decayed *Picea abies* and *Abies sibirica* logs, as well as forest litter both adjacent to and distant from the logs. Compared with forest litter, the wood had higher water content and possessed substantially lower concentrations of heavy metals, which suggests its potential favorability for biota in polluted areas. The pollution-induced loss of fungal diversity in forest litter reached 34% and was stronger in the microhabitats not influenced by CWD. Meanwhile, wood fungal communities lost less than 10% of their total richness and even increased in alpha diversity. These processes led to the diversity and compositional convergence of fungal communities from different microhabitats and substrates in polluted areas. Despite this, the importance of wood and CWD-influenced microhabitats for fungal diversity maintenance was low. Apart from wood-associated fungi, the taxa whose diversity increased in the wood of polluted areas were ectomycorrhizal fungi and eurytopic soil saprotrophs (Mucoromycota, Mortierellomycota, Eurotiomycetes, and Helotiales) that easily tolerate highly toxic litter. Within the majority of pollution-sensitive soil saprotrophic groups, only terricolous Tricholomataceae benefit from CWD as microrefugia. Upon considering the ecological variability within low-rank taxa, the importance of decayed logs as safe sites can be high for certain soil-inhabiting fungal groups in polluted areas.

## Introduction

In natural unmanaged boreal forests, coarse woody debris (CWD) accounts for substantially more than 50% of forest floor detritus mass (Grier and Logan, 1977). It is essential for highly diverse saproxylic biota (Lassauce et al., 2011) and provides food, shelter, and opportunities for reproduction among other ecological guilds (Harmon et al., 1986; Tedersoo et al., 2003, 2008; Koch et al., 2010; Fukasawa, 2012, 2016, 2021; Stokland et al., 2012; Dittrich et al., 2014; Checko et al., 2015).

Fungi are among the principal decomposers and inhabitants of deadwood (Stokland et al., 2012). Until the latest stages of decay, the CWD fungal community is dominated by mycelial wood decomposers accompanied by fungicolous species and yeasts foraging on the products of wood degradation (Baldrian et al., 2016; Mäkipää et al., 2017; Lepinay et al., 2021). Root symbionts and endophytes, predatory fungi, as well as endo- and ectosymbionts of saproxylic arthropods, are revealed in wood through the course of decomposition (Lindahl et al., 1999; Tedersoo et al., 2008; Fukasawa, 2012; Ottosson, 2013; Ottosson et al., 2015; Birkemoe et al., 2018). Upon approaching soil humus in structure and chemical composition, CWD becomes more and more inhabited by saprotrophic soil fungi as well as soil animals and plant roots accompanied by their fungal symbionts (Stokland et al., 2012; Persson et al., 2013; Mäkipää et al., 2017; Vorobeichik et al., 2020).

Influencing the surroundings directly (e.g., due to water retention, shadowing, and leaching) and indirectly (e.g., through the extended mycelium of wood decomposers and activity of wood-dwelling animals), CWD pieces produce around them specific microhabitats essential for certain soil dwellers (Boddy and Watkinson, 1995; Kim et al., 2008) and ameliorate moisture and temperature fluctuations, creating “safety islands” for forest floor biota during periods of extreme weather (Graham, 1925; Jaeger, 1980; Boddy, 1983; Maser and Trappe, 1984; Maser et al., 1988). CWD buffering capability against environmental extremes makes it important for forest biodiversity after tree clearings (Goldin and Hutchinson, 2014) and suggests its vitality in long-term stressed ecosystems (Harmon et al., 1986). For example, it was shown that CWD ameliorates soil acidification in broad-leaved forests subjected to acid precipitation, thereby favoring mollusks in the surrounding forest litter (Kappes et al., 2007).

**FIGURE 1:**
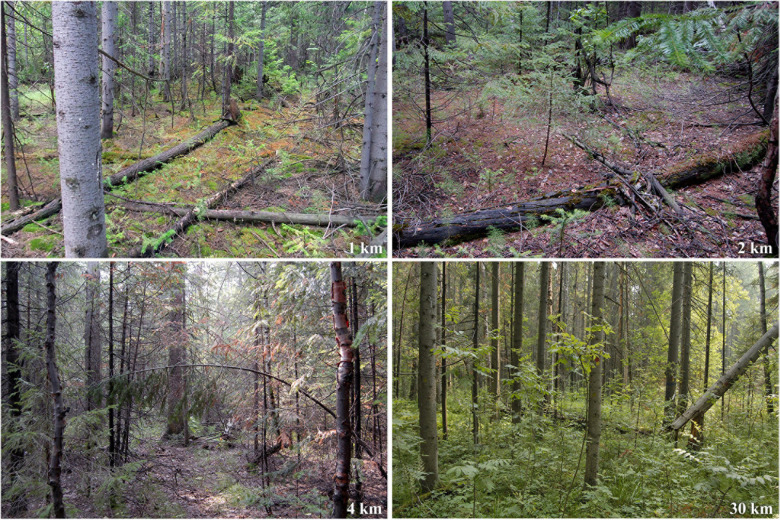
Spruce-fir forests located 1, 2, and 4 km upwind from a large copper smelter in the Central Ural almost completely lack herbaceous layer (the photos are taken at the end of July). Before the smelter was launched 80 years ago, they had been similar to the unaffected forest 30 km from the smelter.

Forests adjacent to large industrial enterprises represent the ecosystems suffering severe long-term stress (Figure 1). They are characterized by altered soil pH and extremely high concentrations of metals in the soil (Dudka and Adriano, 1997; Kozlov et al., 2009). Due to the toxic effect of metals aggravated by high soil acidity, soil fungi and bacteria decline in abundance and diversity close to the sources of pollution (Chen et al., 2014; Mikryukov et al., 2015; Bérard et al., 2016; Mikryukov and Dulya, 2017), while earthworms, enchytraeids, mollusks, and millipedes are almost absent (Bengtsson et al., 1983; Spurgeon and Hopkin, 1996; Vorobeichik, 1998; Vorobeichik et al., 2020). It is reasonable to consider any potential “safety island” mitigating pollution load on biota, maintaining its diversity and functioning, and facilitating its restoration after the load cessation. To our knowledge, there is only one work encountering CWD microrefugial role for soil dwellers, namely, earthworms, in such a strongly damaged ecosystem (Vorobeichik et al., 2020).

To fill this informational gap, we performed the study in one of the best-studied heavily polluted ecosystems in the southern taiga forests of the Urals, which have been affected by a large copper smelter for over 80 years, emitting acid gases and dust with sorbed metals. The smelter annual emissions comprised 150–225 Mt up to 1990, leading to a substantial increase of metal content in adjacent soil and biota. Despite the gradual decrease of the emissions to 2–3 Mt/year, soil pollution levels remain the same (Vorobeichik and Kaigorodova, 2017). Within the first 2–4 km from the chimneys, concentrations of acid-soluble forms of Cu, Pb, Cd, and Zn in soil organic horizons far exceed 3,000, 1,900, 15, and 600 μg/g, respectively (Vorobeichik and Pishchulin, 2011; Vorobeichik and Kaigorodova, 2017; Mikryukov et al., 2020). These levels 5– to 100-fold outreach regional values, registered in the same forests 20–30 km from the smelter. Metal toxicity (enhanced in acidified soil) almost eliminated the herbaceous layer, intensified tree dieback (Vorobeichik and Khantemirova, 1994; Vorobeichik et al., 2014), and strongly reduced the diversity of soil and forest floor invertebrates (Vorobeichik and Bergman, 2020), fungi, and bacteria (Mikryukov and Dulya, 2018). In these areas, fungi of the soil organic horizons decreased in species number by about 30% (Mikryukov and Dulya, 2017, 2018; Mikryukov et al., 2020), demonstrating lower pollution vulnerability than many other studied taxa (arthropods, worms, mollusks, and plants) and raising the question about the potential microrefugia maintaining their diversity. CWD at the latest decomposition stages principally suitable for many soil fungal species and less permeable for pollutants than soil (Dulya et al., 2019) is a perfect candidate for the role of such microrefugia.

This work evaluates the role of CWD for fungal diversity maintenance in spruce-fir forests suffering from long-term industrial pollution. We assume that CWD acts as a microrefugium for soil-inhabiting fungi in polluted areas. It is also hypothesized that the hospitality of forest litter shadowed by CWD is higher than that of litter located far from CWD and increases in polluted areas. The mechanisms of a pollution-induced increase of CWD and its surrounding hospitality can include changes in the microhabitat preferences of particular fungal groups toward CWD and CWD-shadowed forest litter with increasing levels of pollution.

## Materials and Methods

### Sampling

The study was conducted in spruce-fir forests (*Abieto-Piceetum oxalidozum*) surrounding the Middle Ural copper smelter (Revda town, Sverdlovsk Oblast, Russia), which has been operating since 1940. The material was collected in three areas: unpolluted (UP), moderately polluted (MP), and HP areas located 30, 4, and 1–2 km upwind from the smelter, respectively (Supplementary Figure 1A). The areas are as similar as possible in terms of biotope and soil characteristics (Supplementary Table 1); however, they differ strongly in the degree of ecosystem damage caused by pollution (Table 1). To consider forest spatial variability, three sites (2,817–17,153 m^2^ and 100–800 m from each other) were established within each area in September 2017. All autochthonous downed fir (*Abies sibirica* Ledeb.) and spruce [*Picea abies* (L.) H. Karst.] logs of the fourth and fifth decay classes [as per Storozhenko (1990); Supplementary Table 2] were considered for sampling. In each of the nine sites, we randomly selected 10 logs of at least 10-cm base diameter that were in contact with the ground and undamaged by large animals.

**TABLE 1:** Wood and forest litter characteristics in unpolluted (UP), moderately polluted (MP), and heavily polluted (HP) areas.

Parameter	Wood			Forest litter close to CWD			Forest litter far from CWD		
	UP area	MP area	HP area	UP area	MP area	HP area	UP area	MP area	HP area
Number of fir/spruce logs	19/11	12/18	18/12	–	–	–	–	–	–
Moss coverage, % of observed area	90.17 ± 12.21	66.83 ± 25.55	61.07 ± 29.97	–	–	–	–	–	–
Number of logs with herbs/tree seedlings	21/5	16/19	0/18	–	–	–	–	–	–
Circumference, cm	68.50 ± 22.34	68.83 ± 18.7	63.07 ± 28.51	–	–	–	–	–	–
Residence time, years	31.80 ± 12.47b	40.9 ± 13.14a	42.33 ± 11.54a	–	–	–	–	–	–
R_P_, J/cm^3^	3.77 ± 2.03a	2.52 ± 2.14b	3.13 ± 2.21ab	–	–	–	–	–	–
Submergence,%	25.14 ± 18.67b	38.19 ± 15.92a	41.13 ± 19.29a	–	–	–	–	–	–
Moss coverage, %	90.17 ± 12.21a	68.97 ± 22.72b	61.07 ± 29.97b	–	–	–	–	–	–
Moisture content	3.76 ± 1.14b	3.82 ± 1.09b	3.05 ± 0.86a	2.21 ± 0.51	2.14 ± 0.42	2.03 ± 0.49	1.95 ± 0.35	1.95 ± 0.40	1.86 ± 0.47
Ash content, %	2.23 ± 1.18	1.52 ± 0.80	1.66 ± 0.76	21.46 ± 7.47a	13.84 ± 3.45b	19.56 ± 5.58a	21.77 ± 8.33a	15.43 ± 5.43b	21.99 ± 8.69a
Hydrolytic acidity, mkM/100 g	52.05 ± 14.41b	59.63 ± 16.06ab	62.41 ± 11.38a	36.66 ± 7.72c	57.03 ± 6.70b	65.72 ± 6.24a	34.16 ± 9.31c	56.73 ± 6.49b	65.66 ± 7.04a
pH	3.62–5.73a	3.77–5.1ab	3.54–4.81b	4.94–6.35a	4.58–5.38b	4.32–5.33b	4.95–6.65a	4.56–5.45b	4.43–5.53c
P_2_O_5_, mg/100 g	16.78 ± 5.29	15.03 ± 7.65	20.71 ± 15.16	41.06 ± 9.80b	34.34 ± 5.19b	77.62 ± 23.67a	42.43 ± 8.78b	34.03 ± 3.72b	69.27 ± 22.15a
Total nitrogen, %	0.31 ± 0.14	0.22 ± 0.09	0.29 ± 0.17	1.63 ± 0.23	1.68 ± 0.27	1.61 ± 0.24	1.54 ± 0.25	1.69 ± 0.25	1.58 ± 0.27
**Concentrations of acid-soluble metal forms, μg/g**									
Cu	6.32 ± 3.74c	46.02 ± 41.39b	188.13 ± 161.52a	23.50 ± 6.63c	929.80 ± 402.51b	2,406.89 ± 904.42a	22.80 ± 6.17c	959.42 ± 306.07b	2,405.10 ± 883.93a
Zn	48.24 ± 14.23c	125.90 ± 56.44b	240.68 ± 140.54a	232.94 ± 95.50b	574.85 ± 193.29a	554.55 ± 179.37a	193.00 ± 78.53b	560.38 ± 226.88a	537.28 ± 217.76a
Cd	0.53 ± 0.20c	2.06 ± 1.01b	4.27 ± 2.45a	2.12 ± 0.66b	10.73 ± 3.53a	10.63 ± 4.13a	1.92 ± 0.45b	10.19 ± 4.12a	10.04 ± 4.88a
Pb	2.81 ± 2.31c	20.55 ± 18.71b	67.69 ± 58.34a	47.92 ± 20.19c	1,013.41 ± 307.84b	1,959.55 ± 625.77a	42.19 ± 14.97c	1,031.18 ± 276.62b	1,945.26 ± 651.70a
Fe	51.00 ± 49.17b	58.22 ± 59.69b	191.82 ± 188.68a	985.39 ± 380.89b	1,708.40 ± 946.31b	5,856.60 ± 3,463.11a	1,232.81 ± 784.95b	1,894.29 ± 889.79b	5,919.21 ± 4,261.33a
**Concentrations of exchangeable metal forms, μg/g**									
Cu	0.91 ± 0.71c	4.94 ± 6.01b	23.37 ± 24.95a	0.68 ± 0.19c	21.55 ± 11.70b	128.26 ± 74.66a	0.64 ± 0.18c	22.37 ± 10.52b	134.13 ± 96.83a
Zn	23.61 ± 8.84c	75.21 ± 34.03b	137.78 ± 88.23a	32.48 ± 11.98b	203.07 ± 48.23a	264.57 ± 78.57a	24.85 ± 10.14b	195.98 ± 58.37a	240.55 ± 72.23a
Cd	0.40 ± 0.20c	2.17 ± 1.13b	4.13 ± 2.77b	0.81 ± 0.26b	7.85 ± 2.06a	9.29 ± 3.59a	0.74 ± 0.25b	7.53 ± 2.25a	8.26 ± 3.04a
Pb	0.86 ± 0.31c	2.78 ± 2.09b	7.47 ± 8.10a	0.78 ± 0.29c	16.03 ± 9.35b	59.14 ± 26.02a	0.81 ± 0.31c	16.38 ± 8.71b	54.88 ± 30.72a
Fe	4.35 ± 2.84b	5.45 ± 5.21b	16.45 ± 21.70a	1.91 ± 0.93c	18.33 ± 20.88b	95.02 ± 91.89a	2.23 ± 2.55c	21.51 ± 26.39b	103.72 ± 95.63a

*Mean values and standard deviations are shown; minimal and maximal values are provided for pH; *n* = 30 samples. Different letters depict statistically significantly different mean values between areas for a particular substrate (Tukey’s test, α = 0.05). CWD, coarse woody debris.*

We measured the length of each sampled log with a laser distance measurer Disto A3 (Leica Geosystems, St. Gallen, Switzerland). The circumference of the top and base (right above the root collar, if present) was measured using a measuring tape with an accuracy of 0.5 cm. Wood resistance to penetration was measured using a dynamic penetrometer (Larjavaara and Muller-Landau, 2010); the time of tree death was determined through dendrochronological cross-dating; and taxonomic identification was performed through the microscopic inspection of resin canals and wood structure [for details, see Dulya et al. (2019)].

On each log, we marked three sampling points more than 1 m apart from each other (Supplementary Figure 1B and Supplementary Table 3). Within a 15-cm distance from the log, CWD-influenced litter subsamples were collected near each point and pooled. From 1.5 to 15 m from these logs and any other CWD, we collected and pooled three subsamples of CWD-uninfluenced litter (the distance between subsamples was equal to the distance between CWD-influenced subsamples). According to the sampling protocol, loose recently fallen plant debris (OL layer) was gently removed from the sampling point, and approximately 10 g of material was collected from the OF (partly decomposed fragmented material) and OH (well-humified) litter layers using a sterile plastic container. At each marked point on the logs, a disk was cut out with a chainsaw, and 10 g of wood fragments was extracted from each disk with bleach-sterilized forceps and pooled in a sterile plastic container. The samples from each microhabitat type were kept cool and then frozen within 2–8 h for genetic analysis. At the same sampling points, composite samples of 45–780 g of wood or litter were collected for chemical analyses. In further statistical analyses, wood, as well as the CWD-influenced and CWD-uninfluenced litter collected from/near the same log, was treated as belonging to the same sampling plot.

### Physical and Chemical Analyses

In the laboratory, samples intended for chemical analyses were weighed, dried at 65°C for 4 days, and reweighed to determine gravimetric moisture content (MC). Air-dried samples were ground and sieved through 2-mm sieves (MF 10 basic; IKA, Staufen, Germany). Acid-soluble and exchangeable forms of the main metallic pollutants (Cu, Pb, Cd, and Zn) were extracted with 20 ml of 5% HNO_3_ and 0.05 N of CaCl_2_ solutions, respectively, from 1 g of each sample. After 1 h of shaking and 24-h incubation at room temperature, the extracts were filtered through a paper filter with 8- to 12-mm pore size (Melior XXI, Moscow, Russia). Metal concentrations were measured with an atomic-absorption spectrometer AAS Vario 6 (Analytik Jena, Jena, Germany) following the USEPA Method 7000B (USEPA, 2007). Concentrations of P_2_O_5_ (extracted with 0.2 N of HCl) were analyzed with a PE-5400v spectrophotometer (Ecros, Saint Petersburg, Russia). The pH of the wood in distilled water extract (1:25) was measured after 5 min shaking using an inoLab 740 ionometer (WTW, Weilheim, Germany). Total Kjeldahl nitrogen content was measured in 0.5 g of forest litter and 1 g of wood using digestion and distillation units DK 20 and UDK 139 (VELP Scientifica, Usmate Velate, Italy). Ash content (fraction of dehydrated sample) was measured after combustion at 600°C for 6 h in a laboratory furnace (PM-16, Elektropribor, Saint Petersburg, Russia).

### Molecular Analyses

Each frozen sample destined for genetic analysis was tightly enclosed with sterile 4 × 4 gauze dressing and lyophilized. About 6–10 g of air-dried samples was homogenized using coffee grinders sterilized with bleach and UV radiation after each sample. DNA was extracted from sterile-weighted 90- to 105 mg of aliquots using NucleoSpin Soil Kit (MACHEREY-NAGEL, Düren, Germany). The homogenization of aliquots in lysis buffer (SL1, 50 μl of Enhancer SX) was conducted using a Precellys 24 homogenizer (Bertin Instruments, Montigny-le-Bretonneux, France) at 4,000 rpm for 30 s.

We amplified the internal transcribed spacer (ITS2) of rRNA using a two-step PCR protocol following the “16S Metagenomic Sequencing Library Preparation” protocol (Part #15044223 Rev. B, Illumina, San Diego, CA, United States). The primary amplification was done using the primers fITS7 (Ihrmark et al., 2012) and ITS4 (White et al., 1990) with added Illumina adapter overhang nucleotide sequences. The amplicons from the primary PCR were diluted 1:10 in sterile, nuclease-free water; and a second PCR was set up to add the Illumina flow cell adaptors and sample-specific Nextera XT (Illumina) indices. PCR was performed using Tersus high-fidelity DNA polymerase (Evrogen, Moscow, Russia). Amplicons were purified and normalized using the SequalPrep Normalization Plate Kit (Thermo Fisher Scientific, Waltham, MA, United States). All samples were pooled in equimolar amounts, and the size distribution of amplicons was assessed by high-throughput capillary gel electrophoresis (Fragment Analyzer System, Agilent Technologies, Santa Clara, CA, United States). Sequencing was performed on an Illumina MiSeq platform in paired-end mode using a MiSeq Reagent Kit v3 (600 cycles) at Evrogen (Moscow, Russia).

### Bioinformatics Processing and Sequence Identification

Sequencing libraries were demultiplexed using bcl2fastq v2.17.1.14 (Illumina). Primer sequences were trimmed from paired Illumina sequences using cutadapt v.3.1 (Martin, 2011); reads lacking an identifiable primer sequence were discarded. Low-quality reads were filtered out based on the maximum number of expected errors [maxEE > 3; Edgar and Flyvbjerg (2015)] using VSEARCH v.2.15.1 (Rognes et al., 2016). Correction of sequencing errors, paired-end assembly of the forward- and reverse-sequenced reads, and chimera removal using the consensus method were performed with DADA2 v.1.18.0 (Callahan et al., 2016). Denoised sequences were clustered into operational taxonomic units (OTUs) using SWARM v.3.0.0 (Mahé et al., 2021), with the local clustering threshold (*d*) equal to 5 nucleotides. Average within-OTU sequence similarity was 98.3% (Q1 = 97.7, Q3 = 99.2), which is in range with the empirically derived estimates for species hypotheses based on ITS (Kõljalg et al., 2013; Lücking et al., 2020; Tedersoo et al., 2020).

All denoised sequences were taxonomically identified against the curated reference database UNITE v.8.2 (Nilsson et al., 2019) using the hybrid approach of AMPtk v.1.4.1 (Palmer et al., 2018), as follows. Taxonomic assignments were obtained using two methods: global alignment with VSEARCH and a *k*-mer-based SINTAX classifier (Edgar, 2016). SINTAX results were used if taxa displayed a sequence similarity of <97% to the reference database. If sequence similarity was >97%, the result with the finest taxonomic annotation was retained. Post-clustering curation of the OTU table was performed using LULU v.0.1.0 (Frøslev et al., 2017). All steps of the bioinformatics analysis workflow were managed with Snakemake v.5.31.1 (Mölder et al., 2021).

### Statistical Analysis

Operational taxonomic unit counts with taxonomic assignments and associated sample metadata were handled with phyloseq package v.1.32.0 (McMurdie and Holmes, 2013) of R v.4.0.5 (R Core Team, 2021). Data were visualized with ggplot2 v.3.3.3 (Wickham, 2016). Sankey diagrams were constructed using ggalluvial v.0.12.3 (Brunson, 2017).

To account for unequal sequencing depths, we rarefied the data to a depth of 7,689 sequences per sample. Thus, three samples with lower number of reads were excluded from the analyses. For the assessment of community alpha diversity, we used OTU richness (*S*) and effective number of OTUs (*Se*) in a sample (Jost, 2006). As implemented in metagMisc package v.0.0.4 (Mikryukov, 2018), rarefaction was performed 1,000 times; and *S* and *Se* were averaged over the iterations. To avoid index switching artifacts (assignment of reads to incorrect samples during sequencing), prior dissimilarity analysis, we removed the OTUs with a relative abundance of less than 0.25% from each sample (Clavel et al., 2020).

To estimate community dissimilarity, we used Simpson’s index, which is robust to the difference in species richness between communities (Chao et al., 2006). It has an upper limit of one (samples have no OTUs in common) and a lower limit of zero (assemblages are nested – all OTUs from the smaller community are present in a larger community). Ordination was performed with principal coordinates analysis (PCoA) using vegan v.2.5-7 (Oksanen et al., 2020). To examine the differences in community composition, we used non-parametric permutational multivariate analysis of variance [PERMANOVA, 10,000 permutations; Anderson (2001)]. In the test for the difference in community compositions between the substrates, permutations were constrained within an area. The similarity of litter and wood samples in environmental parameters was illustrated with principal component analysis (PCA) using FactoMineR package v.2.4 (Lê et al., 2008).

To compare group means of diversity, similarity, and environmental variables, we used a hierarchical linear model with the substrate, microhabitat type, and pollution area as fixed effects and sampling plot as a random effect (nested within area) to consider the non-independence of individual samples. Concentrations of heavy metals were log-transformed before the analysis. *p*-Values were adjusted for multiple testing by false discovery rate (FDR) correction. The analysis was performed using lme4 v.1.1-26 (Bates et al., 2015). ANOVA and Tukey’s multiple comparison *post hoc* test were conducted using the packages car v.3.0-10 (Fox and Weisberg, 2019) and multcomp v.1.4-16 (Hothorn et al., 2008). To reveal pollution-induced changes in OTU richness, we used Cohen’s *d* effect size (standardized mean difference) and effsize package v.0.8.0 (Torchiano, 2016). Paired Wilcoxon signed-rank test was used to compare CWD-influenced and CWD-uninfluenced litter samples in the numbers of fungal OTUs. Functional annotation of ectomycorrhizal (EcM) taxa was performed using the FungalTraits database v.1.3 (Põlme et al., 2020).

## Results

### Coarse Woody Debris and Forest Litter Parameters

The ratio of sampled fir and spruce CWD within each area mirrored tree stand composition (Table 1 and Supplementary Table 1). Metal concentrations in wood from polluted areas (HP and MP) were significantly higher than in the UP area. CWD from the HP area possessed significantly more P_2_O_5_ but less moisture and lower moss cover. Herbaceous vegetation, mainly represented by *Circaea alpina*, *Linnaea borealis*, *Myosotis sylvatica*, *Oxalis acetosella*, and *Stellaria holostea*, densely covered two-thirds of logs in the UP area but half of the logs in the MP area. Herbs were not observed on the CWD in the HP area, though tree seedlings occupied most logs in both polluted areas (Table 1).

Litter samples of polluted forests were more acidic (ΔpH = 0.99) and had higher metal and P_2_O_5_ concentrations than UP forests (Table 1). The highest ash content in the UP forest litter illustrates its highest mineralization degree. Within each area, heavy metal concentrations in wood were significantly lower than in forest litter, e.g., by four, 20, and 12 times for Cu in UP, MP, and HP areas, respectively. Wood samples were more acidic, less mineralized, and more saturated with water and possessed six times lower nitrogen content than forest litter (Table 1).

**FIGURE 2:**
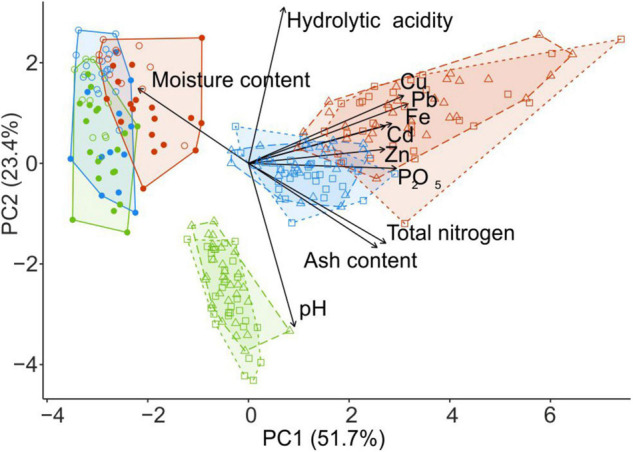
PCA ordination of wood samples collected from fir CWD (filled circles) and spruce CWD (empty circles) and forest litter samples collected close to CWD (triangles) and far from CWD (squares) in the areas with different levels of industrial pollution (green, UP area; blue, MP; red, HP). Arrows denote the relative contribution of each variable to principal components. The percentage on each axis describes the amount of variance explained by principal components. PCA, principal component analysis; CWD, coarse woody debris; UP, unpolluted; MP, moderately polluted; HP, heavily polluted.

Litter close to CWD was moister, slightly but significantly more acidic, and more saturated with Zn and Cd than the litter collected distant from CWD (*F*_1_,_87_ > 6.08, *p* < 0.02). Differences between the substrates and microhabitats are reflected with the samples ordination along two PCA axis in Figure 2.

### Pollution Effect on Forest Litter Fungal Diversity

Illumina sequencing revealed 7,854 fungal OTUs (Supplementary Appendix 1), of which 6,960 (88.6%) were found in litter and 3,908 (49.8%) in wood. The most abundant and diverse phyla were Ascomycota (48.1% of OTUs and 41.4% of reads), Basidiomycota (37.1 and 54.4%), Mortierellomycota (2.3 and 1.5%), and Mucoromycota (1.5 and 0.5%) (Table 2). Other phyla and unclassified fungi comprised 11.0% of OTUs and 1.9% of reads. These five fungal groups were 1.1–3.2 times less diverse in wood than in forest litter.

**TABLE 2:** The diversity and abundance of fungal target groups in different substrates and microhabitats in unpolluted (UP), moderately polluted (MP), and heavily polluted (HP) areas.

Fungal group	Wood			Forest litter close to CWD			Forest litter far from CWD		
	UP area	MP area	HP area	UP area	MP area	HP area	UP area	MP area	HP area
**Total number of OTUs**									
Basidiomycota	609	586	629	1,274	1,075	956	1,291	1,085	915
Ascomycota	833	712	776	1,885	1,436	1,277	1,888	1,368	1,159
Mortierellomycota	63	54	54	104	80	67	93	74	60
Mucoromycota	23	38	24	47	47	41	39	42	40
Other phyla	105	88	80	312	248	255	328	244	231
All phyla	1,631	1,477	1,561	3,620	2,884	2,593	3,637	2,811	2,403
EcM fungi	91	117	99	306	297	272	333	313	286
**OTU richness per sample**									
Basidiomycota	62.1 ± 19.4b	69.2 ± 19.4ab	75.4 ± 18.2a	152.3 ± 28.3a	144.1 ± 22.4ab	130.5 ± 21.2b	148.3 ± 19.7a	141.4 ± 26.6b	118.9 ± 20.8b
Ascomycota	92.8 ± 33.6	86.4 ± 31.8	104.9 ± 26.6	314.1 ± 48.6a	260.0 ± 49.0b	230.8 ± 45.5b	316.7 ± 35.8a	235.8 ± 42.9b	204.8 ± 43.5c
Mortierellomycota	8.7 ± 4.7	9.3 ± 4.3	10.6 ± 3.6	18.8 ± 5.3	19.8 ± 4.7	17.9 ± 3.8	17.3 ± 6.0	17.9 ± 3.6	16.2 ± 3.0
Mucoromycota	1.7 ± 1.9b	4.2 ± 6.2a	2.5 ± 1.7ab	5.1 ± 3.1b	6.7 ± 2.5ab	7.1 ± 3.7a	4.2 ± 2.4b	5.9 ± 2.9ab	6.8 ± 3.6a
Other phyla	6.5 ± 5.5	6.5 ± 3.4	5.9 ± 2.5	34.6 ± 10.9a	24.6 ± 6.9b	28.7 ± 9.6b	36.4 ± 11.8a	24.4 ± 7.7b	25.4 ± 7.1b
All phyla	171.8 ± 59.1	175.7 ± 56.8	199.4 ± 46.2	524.8 ± 80.6a	455.1 ± 69b	415.0 ± 68.5b	523.0 ± 60.1a	425.3 ± 68b	372.2 ± 61.9c
EcM fungi	7.6 ± 4.6b	12.3 ± 5.6a	11.9 ± 4.8a	35.1 ± 7.9b	44.8 ± 10.0a	42.4 ± 8.6a	37.0 ± 8.8b	45.2 ± 11.1a	41.7 ± 9.3ab
**Relative abundance, %**									
Basidiomycota	74.3 ± 20.9	65.0 ± 21.9	69.6 ± 19	45.2 ± 10	52.1 ± 12.7	52.7 ± 10.4	33.3 ± 11.4	48.0 ± 13.5	49.8 ± 10.5
Ascomycota	24.5 ± 20.2	32.4 ± 19.8	27.5 ± 15.9	50.5 ± 10.2	44.0 ± 13.1	41.8 ± 10.3	61.8 ± 12.3	47.0 ± 12.1	43.1 ± 11.3
Mortierellomycota	0.5 ± 0.5	0.9 ± 0.7	1.1 ± 1.2	1.8 ± 1.1	2.2 ± 1.2	2.8 ± 1.8	1.8 ± 1.0	2.5 ± 1.6	3.1 ± 2.4
Mucoromycota	0.2 ± 0.5	1.1 ± 3.0	1.1 ± 5.8	0.2 ± 0.1	0.3 ± 0.6	0.5 ± 0.5	0.2 ± 0.2	0.4 ± 0.7	0.8 ± 2.4
Other phyla	0.5 ± 0.7	0.6 ± 0.8	0.6 ± 0.7	2.3 ± 1.3	1.4 ± 0.9	2.2 ± 2.1	3.0 ± 2.3	2.0 ± 1.1	3.1 ± 5.2
EcM fungi	1.3 ± 1.8	7.5 ± 8.2	15.7 ± 16.8	13.5 ± 9.7	32.6 ± 14.7	37.2 ± 14.3	12.7 ± 7.9	30.1 ± 13.6	36.2 ± 10.9

*Mean ± SD are shown, *n* = 29 samples per area. Different letters depict a statistically significant difference of mean values between areas for a particular substrate revealed with Tukey’s test (*p* < 0.05). CWD, coarse woody debris.*

The total number of OTUs was lower by 17.5% in the MP area and 22.2% in the HP area than in the UP area. The decrease of OTU total number in CWD-uninfluenced forest litter of the MP and HP areas was the strongest of all substrates (22.7 and 33.9%, respectively). OTU richness of four fungal groups declined in polluted areas by 16.0–38.6%, while Mucoromycota increased by 2.6–7.7%.

**FIGURE 3:**
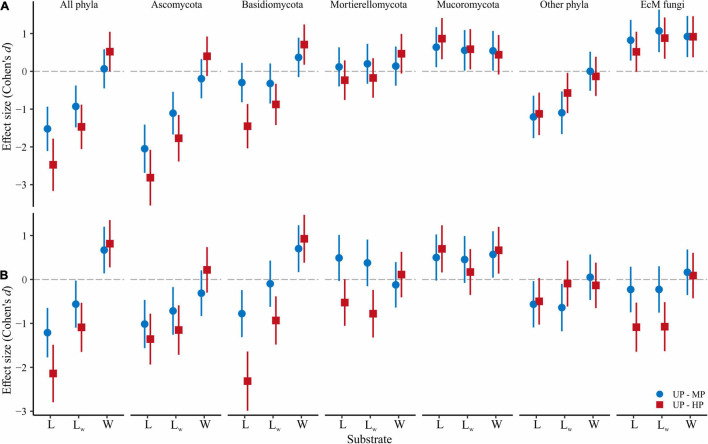
Pollution-induced changes in **(A)** OTU richness and **(B)** effective number of OTUs in MP (circles) and HP area (squares) within three types of forest detritus (L, CWD-uninfluenced litter CWD; L_W_, CWD-influenced litter; W, wood). The negative effect size indicates a pollution-induced decrease of fungal diversity compared with the UP area, positive effect size – diversity increase. Median and 95% CI are shown. OTU, operational taxonomic unit; MP, moderately polluted; HP, heavily polluted; CWD, coarse woody debris; UP, unpolluted.

In CWD-uninfluenced litter, *S* and *Se* were lower in MP area (by 18.7 and 31.3%, respectively) and in HP area (by 28.8 and 52.7%, respectively) than in the UP territory (Table 2 and Figure 3). Basidiomycota and Ascomycota *S* decreased by 4.7–35.3% in polluted areas. Mucoromycota *S* was stimulated by pollution by 40.5 and 61.9% in MP and HP areas, respectively, while Mortierellomycota gained 3.5% of *S* in MP area and lost 6.4% in HP area. Other phyla together with unclassified fungi lost 33.0 and 30.2% of *S* in the MP and HP areas, respectively. *Se* response to pollution was similar to *S* for all target groups (Figure 3B). In accordance with these results, *S* and *Se* of most target groups in litter were negatively affected by acidity, P content, and Cu content (−0.74 < β < −0.28; Supplementary Table 4). In contrast, Mucoromycota *S* and *Se* were positively influenced by litter acidity and Cu concentrations (0.33 < β < 0.49), while Mortierellomycota were not responsive to any of the predictors.

**FIGURE 4:**
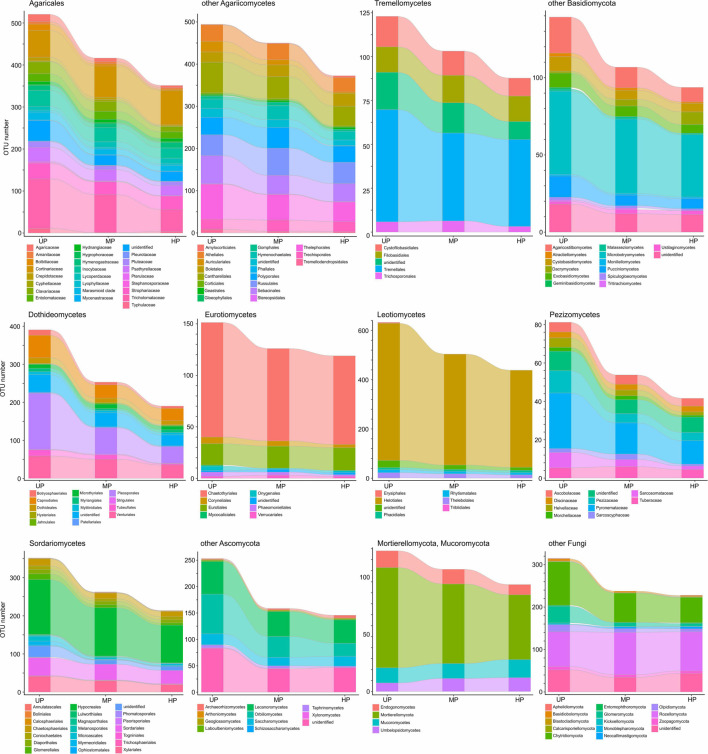
Total OTU number of fungal taxa in the litter collected far from CWD in unpolluted (UP), moderately polluted (MP), and heavily polluted (HP) areas (*n* = 29 samples per area). OTU, operational taxonomic unit; CWD, coarse woody debris.

Based on pollution-induced changes of total OTU number in CWD-uninfluenced forest litter, lower-level taxa were tested for their vulnerability to pollution (Figure 4). Glomeromycota and Olpidiomycota lost most OTUs in polluted areas, while Chytridiomycota diversity decreased by approximately 40%. Of species-rich Ascomycota and Basidiomycota taxa, the most vulnerable (i.e., reduced in diversity by more than half) were Botryosphaeriales, Pleosporales, Tubeufiales, Glomerellales, Microascales, Xylariales, Orbiliomycetes, Agaricaceae, Bolbitiaceae, Clavariaceae, Tricholomataceae, Auriculariales, Agaricostilbomycetes, and Pucciniomycetes. Less vulnerable taxa, which lost 50–20% of OTUs in polluted areas, included Capnodiales, Dothideales, Venturiales, Chaetothyriales, Helotiales, Rhytismatales, Thelebolales, Chaetosphaeriales, Diaporthales, Hypocreales, Sordariales, Pezizomycetes, Lecanoromycetes, Inocybaceae, Marasmioid clade, Pluteaceae, Psathyrellaceae, Cantharellales, Sebacinales, Thelephorales, Cystofilobasidiales, Tremellales, Cystobasidiomycetes, and Microbotryomycetes. Conversely, Microthyriales, Saccharomycetes, Entolomataceae, Strophariaceae, Atheliales, Hymenochaetales, and Polyporales lost <18% of OTUs. Mucorales, Umbelopsidales, Eurotiales, Cortinariaceae, Boletales, Russulales, Trechisporales, and Rozellomycota increased in OTU total number by 3.1–54.8% in MP area and by 0.1–48.9% in HP area. The changes of alpha diversity at lower taxonomic levels were quite similar to the changes revealed for the total OTU richness (Supplementary Figure 2).

The total OTU number of EcM fungi in CWD-uninfluenced litter decreased by 6.0 and 14.1% in MP and HP areas, respectively (Table 2); meanwhile, they increased in alpha diversity by 22.2 and 12.7% and in relative abundance by 2.4 and 2.9 times, exceeding 35% of fungal reads in the litter of the HP area. EcM lineages observed in the litter differed in the sensitivity to pollution. The most notable (2.2–84.4 times) abundance increase in both polluted areas was characteristic of /*boletus*, /*pseudotomentella*, /*endogone*, /*hygrophorus*, /*amphinema-tylospora*, /*suillus-rhizopogon*, /*laccaria*, and /*russula-lactarius* EcM fungal lineages. The abundance of /*paxillus-gyrodon* and /*piloderma* remained unchanged in the MP area and increased in the HP area by 5.1 and 3.0 times, respectively, while /*otidea* abundance increased in the MP area 10.8-fold and remained unchanged in HP area. The /*hydnotrya*, /*tuber-helvella*, /*sebacina*, /*amanita*, and /*inocybe* EcM fungal lineages were relatively non-sensitive to pollution (from 0.7-fold decline to 1.9-fold increase). The most sensitive EcM fungal lineages included /*tomentellopsis*, /*tomentella-thelephora*, /*tricholoma*, /*wilcoxina*, /*rhodoscypha*, /*clavulina*, and /*tarzetta*, which declined 20–90% in the MP area and 33–100% in the HP area.

### Fungal Diversity and Its Maintenance in Wood

**FIGURE 5:**
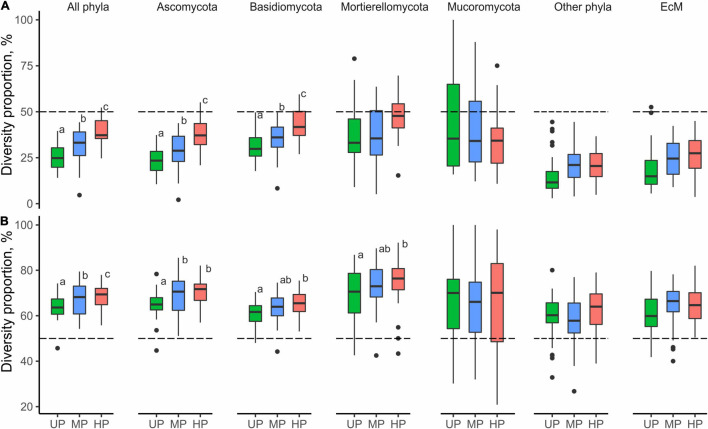
The ratio of OTU numbers revealed in **(A)** wood and **(B)** CWD-influenced litter samples to CWD-uninfluenced litter samples in the areas with different levels of pollution. Different letters indicate statistically significant differences of mean values between areas, revealed with Tukey’s test (*p* < 0.05, *n* = 29; Supplementary Table 6). A few outliers in Mucoromycota are not shown due to clipping of the *y*-axis at the top for readability (however, the analysis was performed on the full dataset). OTU, operational taxonomic unit; CWD, coarse woody debris.

**FIGURE 6:**
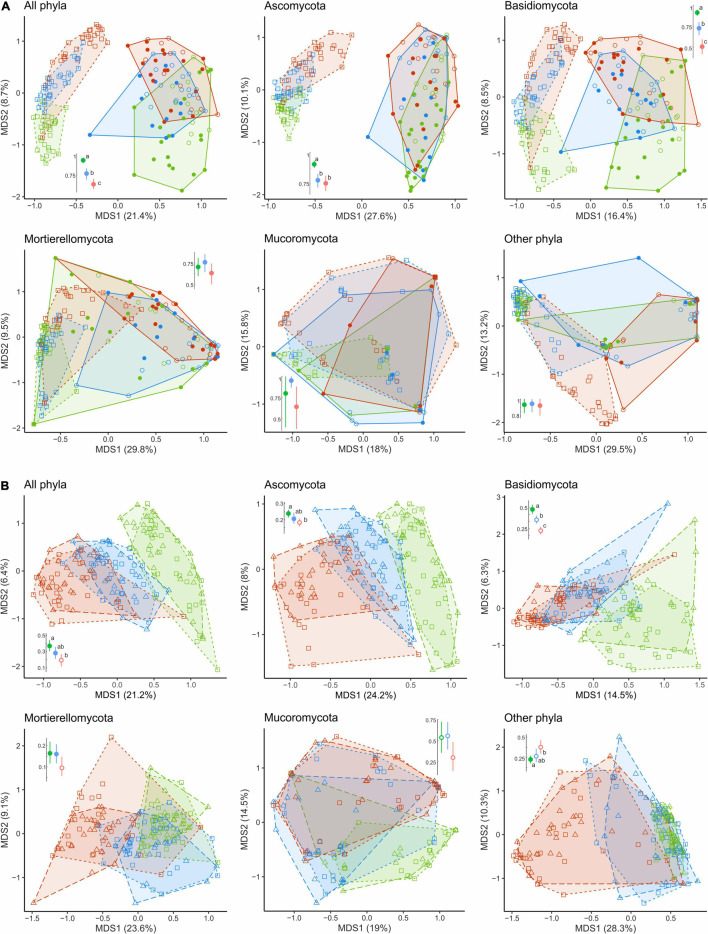
Compositional dissimilarity of fungal communities of CWD-uninfluenced litter from **(A)** wood and **(B)** CWD-influenced litter in the areas with different levels of pollution (green, UP area; blue, MP area; red, HP area). Filled circles denote fir wood; empty circles, spruce wood; squares, forest litter far from CWD; triangles, forest litter close to CWD. Average dissimilarity values with 95% CI are shown next to each ordination diagram: filled dots indicate statistically significant compositional divergence between microhabitats within an area (PERMANOVA test, *p* < 0.05). Different letters indicate significant differences of within-area dissimilarity between the areas (Tukey’s test, *p* < 0.05). CWD, coarse woody debris; UP, unpolluted; MP, moderately polluted; HP, heavily polluted.

The overall OTU richness in the wood from the MP and HP areas was 9.4 and 4.3% lower than in the UP forests. Depending on the fungal group, pollution had no effect or strongly positively affected *S* and *Se* in wood (Table 2, Figure 3 and Supplementary Figure 3). As a result, the ratio of alpha diversity between wood and litter samples significantly increased in the gradient of pollution for all fungi, Ascomycota, and Basidiomycota (Figure 5A). The fungal compositional dissimilarity between wood and forest litter decreased in the pollution gradient (Figure 6A and Supplementary Tables 5, 6), though it remained significant in all groups.

**FIGURE 7:**
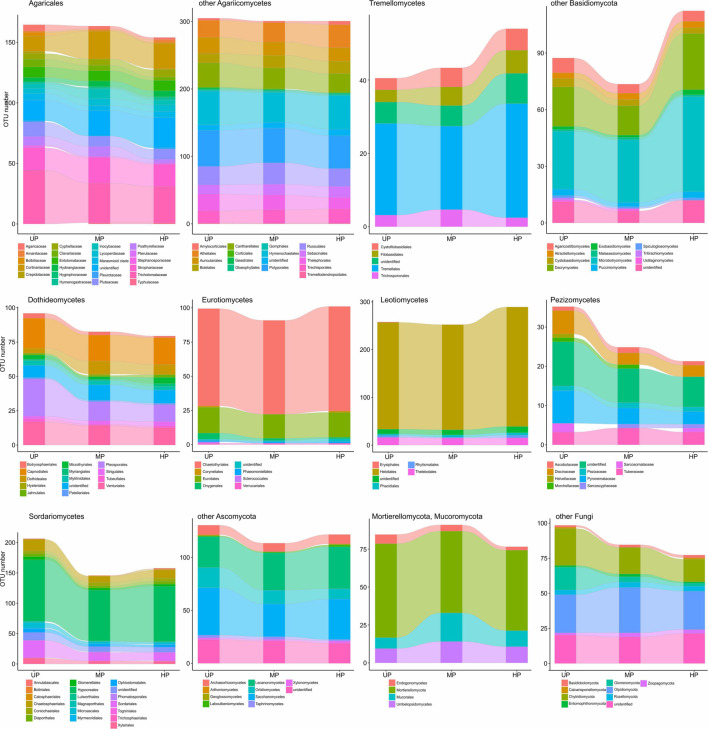
Total OTU number of fungal taxa in the wood from unpolluted (UP), moderately polluted (MP), and heavily polluted (HP) areas (*n* = 29 samples per area). OTU, operational taxonomic unit.

Of OTU-rich taxa, total OTU richness of Sordariales, Pleosporales, and Glomeromycota decreased in the wood of polluted areas by more than 50% (Figure 7), whereas Pluteaceae, Tricholomataceae, Thelephorales, Chaetosphaeriales, Venturiales, Orbiliomycetes, and Chytridiomycota decreased by 50–25%. A slightly detrimental effect of pollution on OTU number was observed for Auriculariales, Cantharellales, Polyporales, Saccharomycetes, Capnodiales, Thelebolales, Hypocreales, and Mortierellomycota. Richness of Cortinariaceae, Strophariaceae, Atheliales, Boletales, Hymenochaetales, Russulales, Sebacinales, Trechisporales, Tremellales, Dacrymycetes, Microbotryomycetes, Lecanoromycetes, Chaetothyriales, Eurotiales, Helotiales, and Rozellomycota was increased by pollution.

**FIGURE 8:**
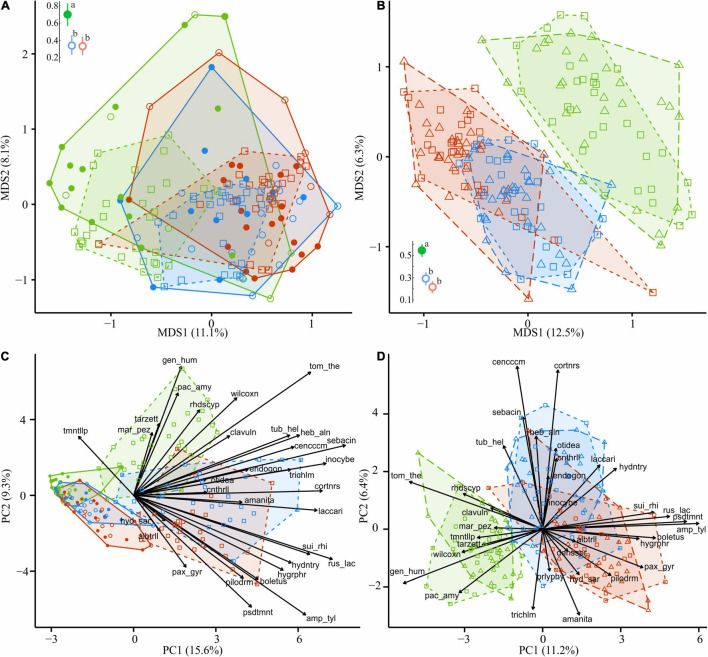
Compositional dissimilarity of EcM fungi from the areas with different levels of industrial pollution (green, UP area; blue, MP area; red, HP area). Filled circles in **(A,C)** denote fir wood; empty circles, spruce wood. Triangles in **(B,D)** denote CWD-influenced litter. CWD-uninfluenced litter is shown with squares. Average dissimilarity values with 95% CI are shown next to ordination diagrams in **(A,B)** filled dots indicate statistically significant compositional divergence between microhabitats within an area (PERMANOVA test, *p* < 0.05); different letters indicate significant effect of area on dissimilarity (Tukey’s test, *p* < 0.05). The arrows indicate the increase in EcM lineage relative abundance toward an area and a substrate. EcM, ectomycorrhizal; UP, unpolluted; MP, moderately polluted; HP, heavily polluted; CWD, coarse woody debris.

In all areas, wood harbored 2.5–3.7 times less EcM OTUs than forest litter (Table 2). EcM total OTU number increased in the wood of the MP area by 28.8% and in the HP area by 8.8%. EcM *S* increased more than twice, while *Se* remained unchanged, showing higher dominance of communities in polluted areas (Figure 3). This guild increased in the relative abundance in polluted wood (Table 2), leading to a decreased ratio of their abundance between litter and wood from 9.8 in the UP area to 4.0 and 2.3 in the MP and HP areas, respectively. Accordingly, EcM fungal compositional dissimilarity between wood and litter declined with the increasing pollution and became non-significant in polluted areas in contrast to other target groups (Figure 8A). The /*boletus*, /*amphinema-tylospora*, /*pseudotomentella*, /*laccaria*, /*paxillus-gyrodon*, /*piloderma*, and /*russula-lactarius* lineages, which showed high tolerance to pollution in the litter, also increased in abundance in polluted wood by 1.7–54.3 times (Figure 8C). Moderately tolerant to pollution, /*inocybe*, */amanita*, /*sebacina*, */otidea*, and /*cortinarius* also increased in polluted wood 2.8- to 83.4-fold. Of EcM fungal lineages negatively responding to polluted litter, /*clavulina*, /*genea-humaria*, /*pachyphloeus-amylascus*, /*tomentellopsis*, and /*tricholoma* suffered a 54–100% abundance decrease in polluted wood. Of the pollution-sensitive lineages, /*wilcoxina* increased in abundance in wood of the MP and HP areas by 10.0 and 8.4 times, respectively, while /*tomentella-thelephora* abundance was comparable with UP values. Notably, the sensitive lineages increased in abundance in dead wood due to certain pollution-tolerant species, such as *Thelephora terrestris* and *Trichophaea* spp.

Based on the difference of OTU occurrences between CWD-uninfluenced litter and wood, a few taxa significantly changed substrate preferences in polluted areas. Chytridiomycota, Dothideales (mainly due to Dothioraceae and Dothideaceae), Pezizales, Lecanoromycetes, Phacidiales, Mucoraceae, and Tricholomataceae became more frequent in wood; while Microthyriales, Strigulales, Chaetosphaeriales, Chaetomiaceae, Aspergillaceae, Piskurozymaceae, Boletales, Hydnangiaceae, and Physalacriaceae shifted toward forest litter (Supplementary Table 7).

Fungal alpha diversity was weakly influenced by within-area physicochemical variability of the substrates (Supplementary Table 8). In the UP area, only litter pH significantly negatively affected *S* of all fungi, Mortierellomycota, and the group “other phyla” (*B* = −89.8, *B* = −9.0, *B* = −19.7, respectively); Mucoromycota diversity negatively responded to litter moisture (*B* = −4.71 and *B* = −2.4 for *S* and *Se*, respectively) in HP area; and Mortierellomycota *S* positively responded to N concentration in wood of the MP area (*B* = 20.4, *p* < 0.05).

### Diversity Maintenance in Forest Litter Shadowed by Coarse Woody Debris

In the UP area, the total number of OTUs close to CWD was 0.5% (17 OTUs) lower than far from CWD due to all groups except for Mortierellomycota and Mucoromycota (Table 2). In polluted areas, CWD-influenced microhabitats’ contribution to fungal diversity in litter increased for most target groups, though remained unchanged for Mortierellomycota and decreased for Mucoromycota. As a result, CWD shadows harbored 2.6 and 7.9% more fungal OTUs in the MP and HP areas, respectively. Notably, the total number of EcM fungal OTUs in polluted areas remained higher in CWD-free litter. However, this difference decreased in the pollution gradient from 8.1% in the UP area to 4.9% in the HP area.

At the level of alpha diversity, CWD-influenced litter samples harbored higher *S* (*Z* = 0.32, *n* = 87, *p* = 0.003), which was ascribed to all Basidiomycota, Ascomycota, Mortierellomycota, and Mucoromycota (*Z* > 0.23, *n* = 87, *p* < 0.03; Supplementary Table 9). EcM fungal *S* was comparable between the microhabitats (*Z* = 0.004, *n* = 87, *p* = 0.97). Pollution-induced decrease of *S* and *Se* in CWD-influenced litter was less pronounced than in CWD-uninfluenced litter: 13.3% for *S* and 20.3% for *Se* in the MP area, and 20.9% for *S* and 37.4% for *Se* in the HP area. Therefore, the relative hospitability of CWD-shadowed microhabitats tended to increase in the gradient of pollution (Figure 5B). However, the effect of area type on the diversity ratio was non-significant, formally rejecting our third hypothesis.

In the UP area, most fungal groups (except Mucoromycota) significantly differed in composition between the CWD-influenced and CWD-uninfluenced litter (Figures 6B, 8B, D). In the MP area, only Ascomycota and Mortierellomycota contributed to the compositional differences between the microhabitats. None of the groups differed compositionally between the two microhabitats in the HP area.

**FIGURE 9:**
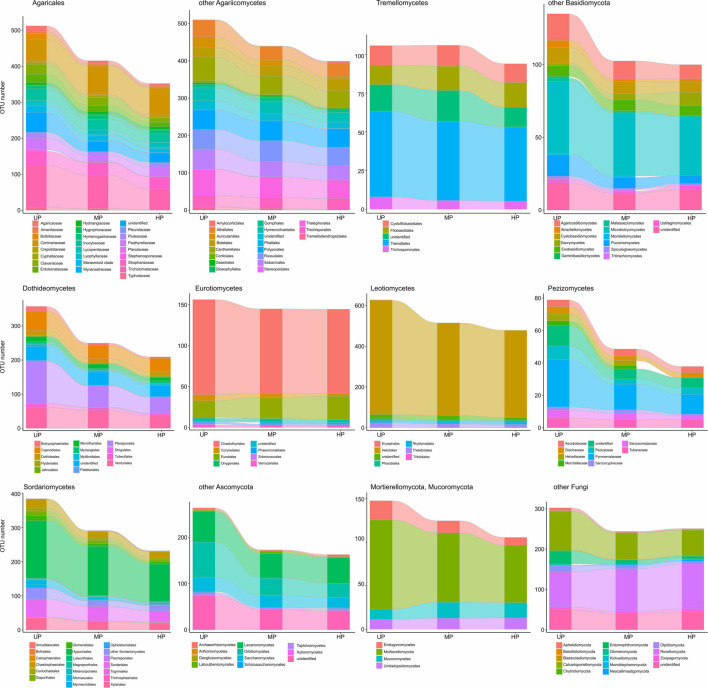
Total OTU number of fungal taxa in the litter collected close to CWD in unpolluted (UP), moderately polluted (MP), and heavily polluted (HP) areas (*n* = 29 samples per area). OTU, operational taxonomic unit; CWD, coarse woody debris.

At low taxonomic levels, pollution-induced changes of total OTU richness and alpha diversity in CWD-influenced forest litter resembled the patterns observed in CWD-uninfluenced forest litter (Figure 9 and Supplementary Figure 4). The exception was a smaller (compared with CWD-uninfluenced microhabitats) decrease in the diversity of Agaricaceae, Psathyrellaceae, Helotiales, Eurotiomycetes, and Mucorales and primarily wood-associated taxa from Tremellomycetes, Lecanoromycetes, Dothideales, Capnodiales, and Xylariales.

The test for pollution-induced changes in microhabitat preferences of OTUs inhabiting the UP and polluted areas showed that only Crepidotaceae became significantly more frequent in the localities close to CWD in the HP area (Supplementary Table 7).

## Discussion

### Fungal Diversity in Litter

The fungal diversity in forest litter decreased in the gradient of pollution, which corroborates the results of soil fungi research in the vicinity of this and other similar enterprises (Liao and Xie, 2007; Wang et al., 2007; Blagodatskaya et al., 2008; Hui et al., 2012; Bérard et al., 2014, 2016; Chen et al., 2014; Mikryukov and Dulya, 2017, 2018). In addition to heavy metal toxicity, the nearly complete elimination of herbaceous vegetation (Trubina, 2009, 2020) likely played an essential role in the impoverishment of fungal communities, which was exemplified by the substantial loss of biotrophic and saprotrophic species associated with herbaceous plants and their litter. In concordance with previous results (Baldrian, 2010; Torres-Cruz et al., 2018; Mikryukov et al., 2020), eurytopic soil saprobes (e.g., Mortierellomycota, Mucoromycota, Eurotiomycetes, and Helotiales) exhibited high tolerance to soil pollution with heavy metals. Unexpectedly, EcM fungi increased in diversity and doubled in relative abundance in polluted litter. Notably, Agaricomycetes representatives are primarily responsible for this effect, while EcM Pezizomycetes (except for *Tuber* spp., *Hydnotrya* spp., and *Otidea leporina*) are as sensitive to pollution as most saprotrophic representatives of this class. The pollution-induced stimulation of EcM fungi supports previous findings of increased EcM abundance in the upper soil horizons of the studied polluted areas (Veselkin, 2004).

We found subtle but systematic differences between abiotic parameters in CWD-shadowed and CWD-free litter. Subtle differences in pH, microclimate, and the proportion of plant detritus fractions between CWD-shadowed and CWD-free microhabitats were previously shown in coniferous, beech, and eucalypt forests and proved to be influential for mollusks, invertebrates, and microbial biomass (Marra and Edmonds, 1998; Evans et al., 2003; Spears et al., 2003; Kappes et al., 2007; Goldin and Hutchinson, 2015). CWD-shadowed litter harbored a distinct composition and higher alpha diversity of fungal communities. However, these results must be treated with caution because lichenized and wood-decomposing fungi, yeasts known from xylophagous arthropods’ frass, as well as the parasites of lichens, polypores, and myxomycetes, were more frequently identified in CWD-shadowed litter than those far from CWD, implying that mycelium, propagules, and dead remnants raining down from wood have a strong positive influence on nearby soil biodiversity estimates. The direct link between fungal communities in wood and underlying litter was also confirmed by a 20% lower compositional dissimilarity between wood and litter samples collected from the same sampling plot when compared with samples collected from different sampling plots (Supplementary Table 10).

Along with pollution load, the contribution of CWD-shadowed microhabitats to litter fungal diversity increased. Mucoromycota, Mortierellomycota, and most soil saprotrophic orders from Eurotiomycetes and Leotiomycetes possessed higher average numbers of OTUs in the CWD-influenced litter of all areas. With the “fungal” rain from CWD, these pollution-tolerant groups could lead to an overestimation of the capacity of CWD-shadowed litter to maintain fungal gamma and alpha diversity in polluted areas. The expansion of pollution-tolerant EcM and saprotrophic fungi also explains the pollution-induced compositional convergence of communities from CWD-influenced and CWD-uninfluenced microhabitats. Additionally, the leaf and root pathogens and symbionts of herbaceous plants, which were more frequent in CWD-free microsites in UP forests, contributed to the compositional and diversity differences between the microhabitats in those areas. The decline of this group, which was caused by the toxicity-induced elimination of potential hosts in polluted areas (Trubina, 2009, 2020), may have led to the convergence of these two microhabitat types.

### Fungal Diversity in Coarse Woody Debris

Notably, there were two to three times more fungal OTUs in the forest litter than in wood. This proportion corresponds to the results obtained from a similar sampling scheme (Mäkipää et al., 2017). The distinct composition of wood fungal communities and the diversity gap between wood and litter indicate that the unintentional influx of DNA from the outside into a piece of CWD is very limited at this decomposition stage.

Considering the low saturation of wood with heavy metals and its high water-holding capacity, we predicted that the impoverishment of fungal communities in CWD would be lower than in HP forest litter. Conversely, the fungal alpha diversity in wood from the moderately polluted area was similar to that of the unpolluted area and increased in HP forests, leading to a higher proportion of forest floor fungal diversity being supported by CWD. One of the primary factors of wood dwellers’ welfare is the availability of woody debris as forage or habitat (Heilmann-Clausen and Christensen, 2003; Bässler et al., 2010; Josefsson et al., 2010; Runnel and Lõhmus, 2017; Runnel et al., 2021). Therefore, the increased tree dieback and treefall caused by pollution close to the smelter (Vorobeichik et al., 2014) could promote fungal diversity in wood. This effect can hardly be neutralized by the observed levels of toxicity in wood. According to metal-tolerance tests, fungi can withstand more than 25 μg/g of water-soluble copper salts (Englander and Corden, 1971; Colpaert and Van Assche, 1987; Daniel and Nilsson, 1988; Woodward and De Groot, 1999). According to the results of chemical analyses, in the heavily polluted area, only one-third of wood samples with 26.6–87.6 μg/g of exchangeable copper forms (which include water-soluble forms) could exceed this threshold, while 90% of forest litter samples contained 28.0–422.0 μg/g of exchangeable copper compounds.

The pollution-induced convergence of fungal diversity and composition between forest litter and wood provide indirect evidence of CWD sheltering soil fungi in polluted areas. However, in addition to typical wood dwellers, the taxa that did not decrease (or even increased) in diversity and relative abundance in polluted wood were the eurytopic soil saprobes and EcM fungi that tolerate polluted soil. Decayed wood can hardly be vital for these groups close to the smelter.

By contrast, saprotrophic Agaricomycetes suffered from a heavy pollution-induced diversity decline in forest litter (as was shown in this and previous works for southern taiga and pre-forest-steppe pine-birch forests; Mikryukov et al., 2020). Therefore, the revealed significant repartition of surviving Tricholomataceae OTUs between wood and forest litter may indicate that CWD can serve as one of the last harbors for this vulnerable group in polluted environments. We focused on saprotrophic members of this taxon and, by using publicly available data on fruiting body observations, classified 88 OTUs annotated to the species level by substrate specialization into terricolous, wood-dwelling, and wood-terricolous species (fruiting on soil, wood, or both substrates, respectively; Supplementary Table 11). The former and latter groups significantly repartitioned toward the wood in the heavily polluted area (Supplementary Figure 5) due to the disproportionate decrease in occurrence within polluted litter and wood. This indicates a sheltering function of CWD in inhospitable environments, even for species considered terricolous. Thus, a peculiar delimitation of species based on substrate requirements provided the opportunity to discern the importance of CWD for non-wood-dwelling fungi. It would be informative to perform a similar analysis of multispecies groups, which repartitioned toward wood under pollution effects (i.e., Pezizomycetes and Lecanoromycetes).

Some of these findings contribute to knowledge about the ecological requirements of poorly studied fungal groups. For instance, we registered Archaeorhizomycetes in a large number of CWD pieces (including in the HP area), which supports their single observation in wood by Rajala et al. (2011) and verifies that Archaeorhizomycetes tend to inhabit acidic and organic-rich substrates (Pinto-Figueroa et al., 2019). Many *Mortierella* OTUs were abundant and frequent in wood. Of these, *Mortierella gemmifera*, *Mortierella pulchella*, and *Mortierella humilis* have already been found in well-decayed Norway spruce CWD (Baldrian et al., 2016). Here, we show that for *Mortierella fimbricystis*, *M. gemmifera*, *Mortierella jenkinii*, and *M. pulchella*, wood can be preferential and thus a potentially important substrate in dark coniferous forests (Supplementary Figure 6).

### Limitations

This work has two obvious limitations regarding replication. First, our work was performed in the vicinity of a single copper smelter and may not be indicative of other heavy metal pollution sources in boreal and temperate forests. However, the patterns of forest litter fungal response to industrial pollution revealed in our previous work in the southern taiga near the Middle Ural copper smelter and birch-pine forests near the Karabash copper smelter (Southern Urals, pre-forest-steppe subzone) are largely similar (Mikryukov et al., 2020). Second, our study plots were geographically aggregated to the source of pollution by distance, which limits their statistical independence and adds an area effect that confounds the pollution effect.

## Conclusion

Our results confirm the vital role of coniferous CWD for boreal forest biodiversity by showing that one-third of terrestrial fungal richness resides in decaying wood. We also found that CWD-shadowed litter has specific physicochemical parameters and may hold a higher number of species than the litter in CWD-free localities. The fraction of species harbored by wood substantially increased in the pollution gradient, which formally verifies the hypothesis that CWD – which is less impregnated with toxicants than forest litter – acts as microrefugium for forest floor fungi. However, our substantive analysis showed that both litter and woody debris are invaded by pollution-tolerant litter dwellers in polluted areas, thereby causing an overestimation of the role of CWD as a microrefugium. The repartitioning of pollution-sensitive fungal orders and families from highly toxic litter to wood appeared to be the most promising tool for CWD importance assessment. However, species’ substrate preferences must be considered to achieve valid inferences. An important implication of the present work is that CWD will hardly provide propagules to facilitate soil fungal community regeneration following toxic load cessation. Thus, the influx of propagules from unpolluted areas through forest corridors must be provided.

## Data Availability Statement

The datasets presented in this study can be found in online repositories and Supplementary Material. DNA sequences recovered in this study were deposited in NCBI Sequence Read Archive (SRA) under accession numbers SRR14752847 – SRR14753115 (BioProject PRJNA735619, Study ID SRP323118, BioSample IDs SAMN19591351 – SAMN19591619). OTU sequences and their occurrences were deposited in a publicly accessible repository at PlutoF platform (https://plutof.ut.ee/).

## Author Contributions

VM and OD designed the study and performed the statistical analyses. VM, OD, and IB collected the samples. OD and AL performed the chemical analysis and functionally annotated Tricholomataceae OTUs. GL performed the molecular-genetic analysis. OD, VM, and LT wrote the manuscript with input from other authors. All authors contributed to manuscript revision and read and approved the submitted version.

## Conflict of Interest

The authors declare that the research was conducted in the absence of any commercial or financial relationships that could be construed as a potential conflict of interest.

## Publisher’s Note

All claims expressed in this article are solely those of the authors and do not necessarily represent those of their affiliated organizations, or those of the publisher, the editors and the reviewers. Any product that may be evaluated in this article, or claim that may be made by its manufacturer, is not guaranteed or endorsed by the publisher.

## Supplementary Material

Supplementary Figure 1 **(A)** Areas with different levels of pollution from the smelter (UP, unpolluted area; MP, moderately polluted; HP, heavily polluted). Dots indicate sites with 10 sampling plots each. **(B)** The scheme of a sampling plot; w_1_–w_3_ depict wood sampling points, L_*w1*_– L_*w3*_, forest litter sampling points in CWD-influenced microhabitats, L_1_–L_3_, forest litter sampling points in CWD-uninfluenced microhabitats. In the upper right corner of the picture, five dots on a woodblock denote the spots from which wood was collected.

Supplementary Figure 2 The average number of fungal OTUs in the litter collected far from CWD in unpolluted (UP), moderately polluted (MP), and heavily polluted (HP) areas (*n* = 29 samples per area).

Supplementary Figure 3 The average number of fungal OTUs in the wood in unpolluted (UP), moderately polluted (MP), and heavily polluted (HP) areas (*n* = 29 samples per area).

Supplementary Figure 4 The average number of fungal OTUs in the litter collected close to CWD in unpolluted (UP), moderately polluted (MP), and heavily polluted (HP) areas (*n* = 29 samples per area).

Supplementary Figure 5 The proportion of occurrence of saprotrophic Tricholomataceae OTUs with different substrate specialization (T, terricolous; WT, wood-terricolous; W, wood-dwelling) in the wood in polluted areas. Comparisons of **(A)** OTU sets inhabiting UP (green) and MP (blue) areas and **(B)** OTU sets inhabiting UP (green) and HP (red) areas. Asterisks denote a significant difference in the fraction of OTUs occurrences in wood between two areas (GLMM, *p* < 0.05).

Supplementary Figure 6 The occurrence of *Mortierella* species in wood, CWD-influenced, and CWD-uninfluenced forest litter.

Supplementary Table 1 Soil and vegetation characteristics in the studied areas. Means ± standard deviations are shown for stand density.

Supplementary Table 2 Storozhenko’s classification system of downed CWD.

Supplementary Table 3 Sample list with SRA accession numbers.

Supplementary Table 4 Relationship between fungal alpha diversity and environmental parameters along the pollution gradient.

Supplementary Table 5 The fungal compositional dissimilarity between different substrates (see Figure 6).

Supplementary Table 6 The effect of area and sampling locality on Simpson’s abundance-based dissimilarity of fungal communities between microhabitats (forest litter close to CWD and far from CWD).

Supplementary Table 7 Occurrence of fungal OTUs in different substrates.

Supplementary Table 8 Relationship between fungal alpha diversity and environmental parameters within each pollution area and substrate type.

Supplementary Table 9 The effect of area on the fungal OTU richness between the substrates or microhabitats.

Supplementary Table 10 Average Simpson’s dissimilarity between wood and forest litter samples collected from different and similar sampling plots.

Supplementary Table 11 Occurrence of Tricholomataceae OTUs with different substrate specialization (defined based on literature data) in wood and forest litter.

Supplementary Appendix 1 Sequences of the fungal ITS2 OTUs observed in the current study (FASTA format).
