# Supplementary material for: Sheltering Role of Well-Decayed Conifer Logs for Forest Floor Fungi in Long-Term Polluted Boreal Forests
Source: Front Microbiol. 2021 Oct 6;12:729244. doi: 10.3389/fmicb.2021.729244 (PMC8527098; doi:10.3389/fmicb.2021.729244)
Supplement: Supplementary Figure 1 — (A) Areas with different levels of pollution from the smelter (UP, unpolluted area; MP, moderately polluted; HP, heavily polluted). Dots indicate sites with 10 sampling plots each. (B) The scheme of a sampling plot; w1–w3 depict wood sampling points, Lw1– Lw3, forest litter sampling points in CWD-influenced microhabitats, L1–L3, forest litter sampling points in CWD-uninfluenced microhabitats. In the upper right corner of the picture, five dots on a woodblock denote the spots from which wood was collected. [file Data_Sheet_2.PDF]

Sheltering role of well decayed conifer logs for forest floor fungi in long-term polluted areas

Vladimir S. Mikryukov, Olesya V. Dulya, Igor E. Bergman, Georgiy A. Likhodeevskii, Anzhelika D. Loginova, Leho Tedersoo

(A)

(B)

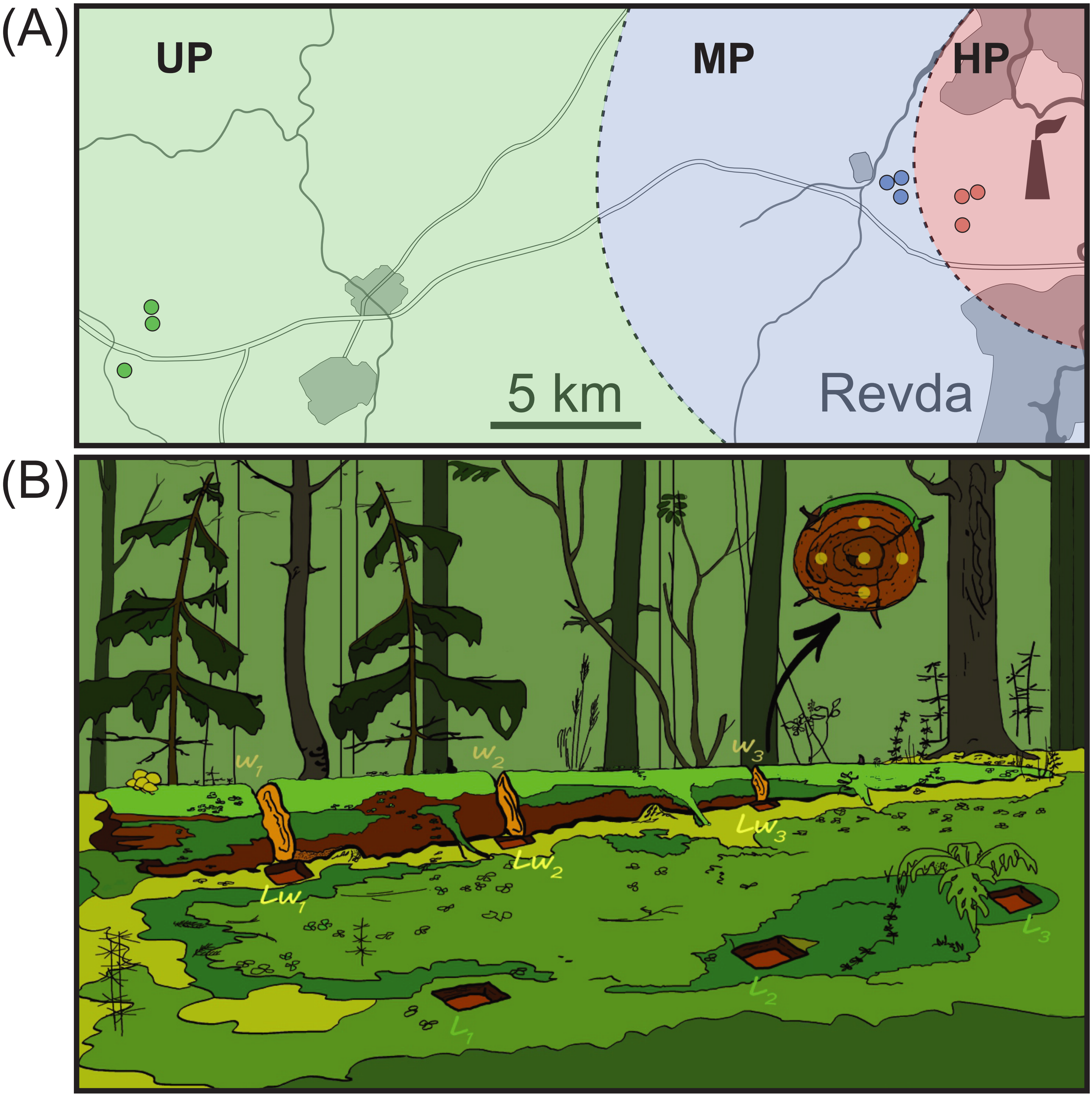

Figure S1. (A) Areas with different levels of pollution from the smelter (UP, unpolluted area; MP, moderately polluted; HP, heavily polluted). Dots indicate sites with 10 sampling plots each. **(B)** The scheme of a sampling plot; w_1–w_3 depict wood sampling points, L_{w1}– L_{w3}, forest litter sampling points in CWD-influenced microhabitats, L_1–L_3, forest litter sampling points in CWD-uninfluenced microhabitats. In the upper right corner of the picture, five dots on a woodblock denote the spots from which wood was collected.

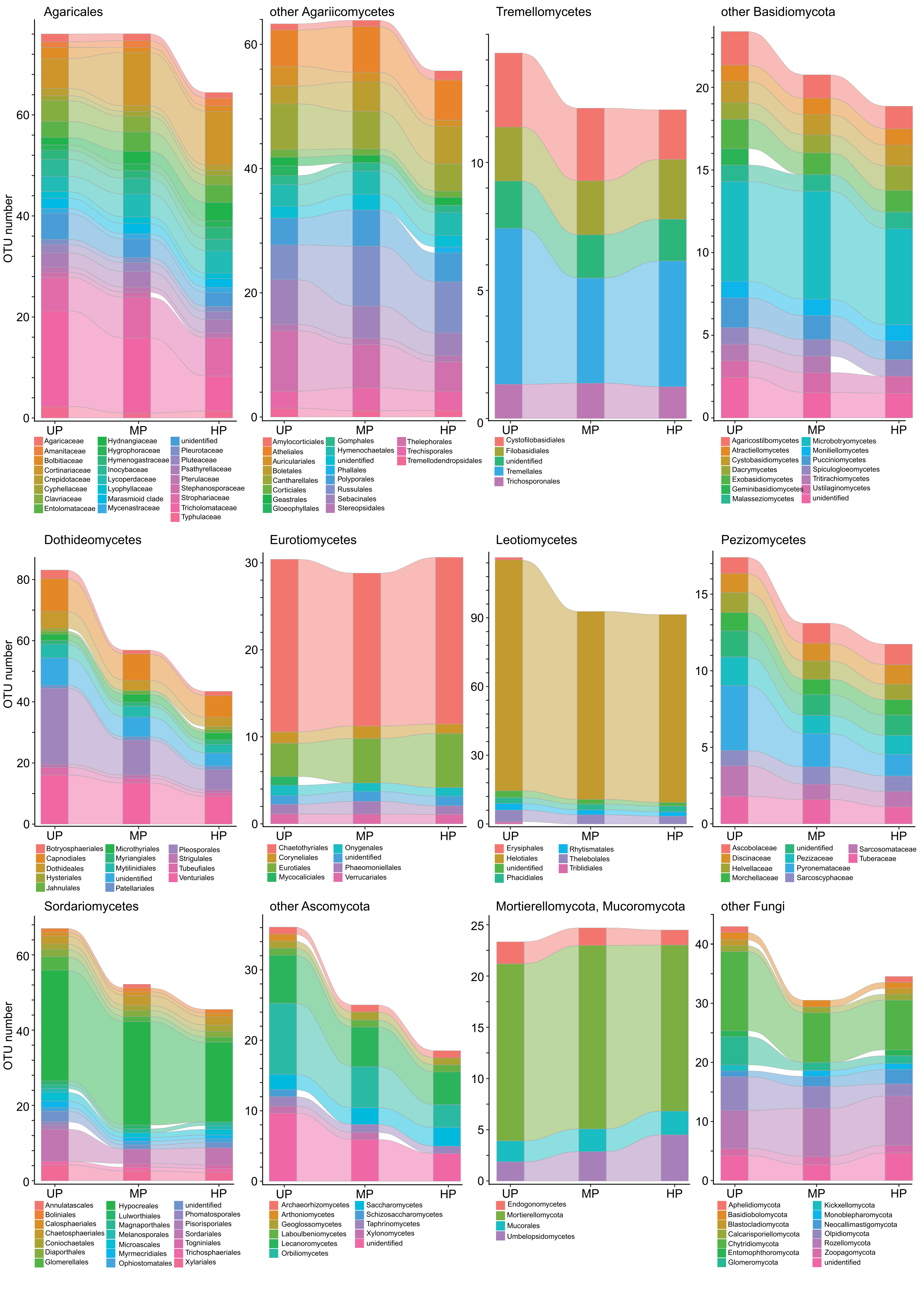

Figure S2. The average number of fungal OTUs in the litter collected far from CWD in unpolluted (UP), moderately polluted (MP), and heavily polluted (HP) areas (n = 29 samples per area).

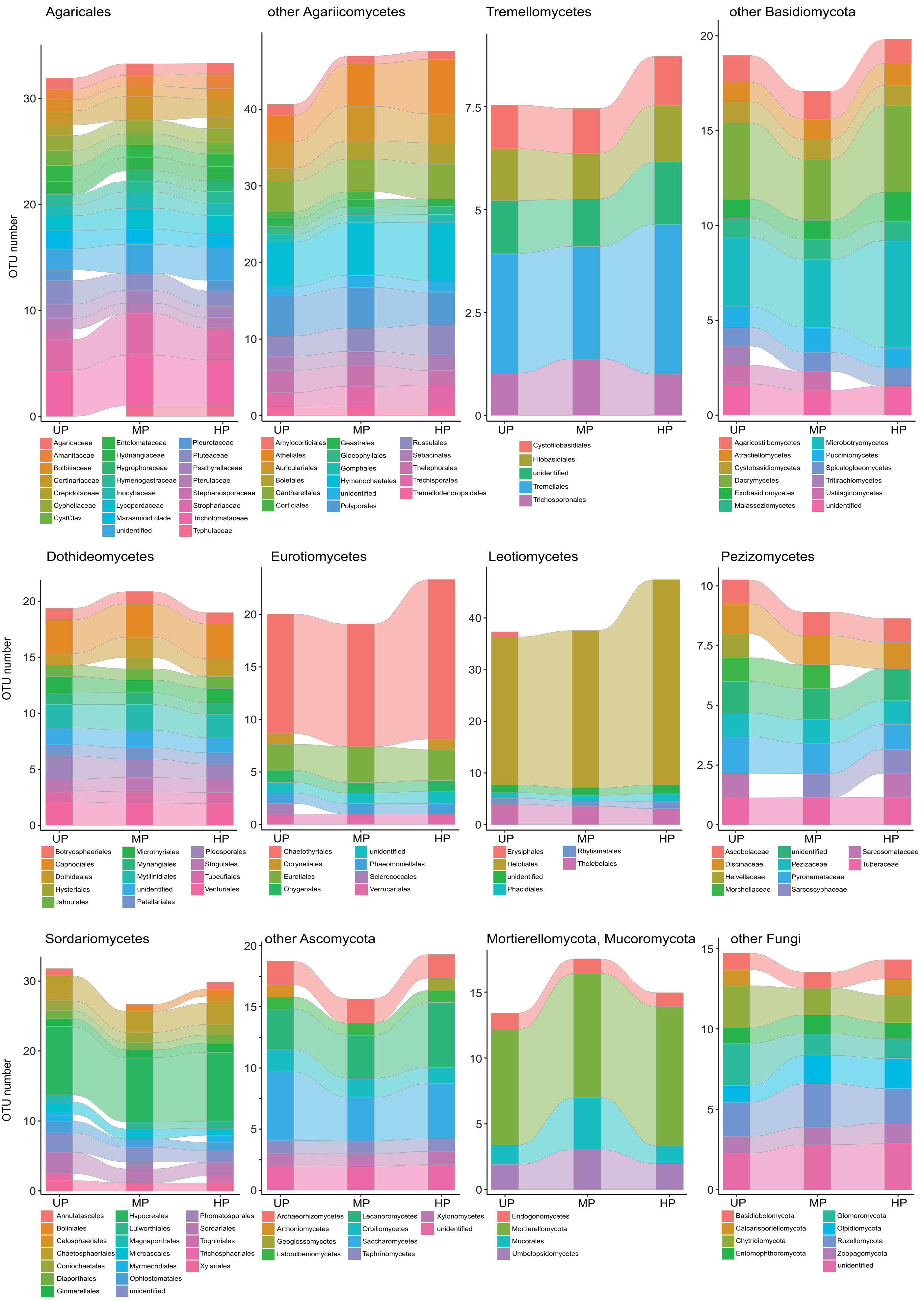

Figure S3. The average number of fungal OTUs in the wood in unpolluted (UP), moderately polluted (MP), and heavily polluted (HP) areas (n = 29 samples per area).

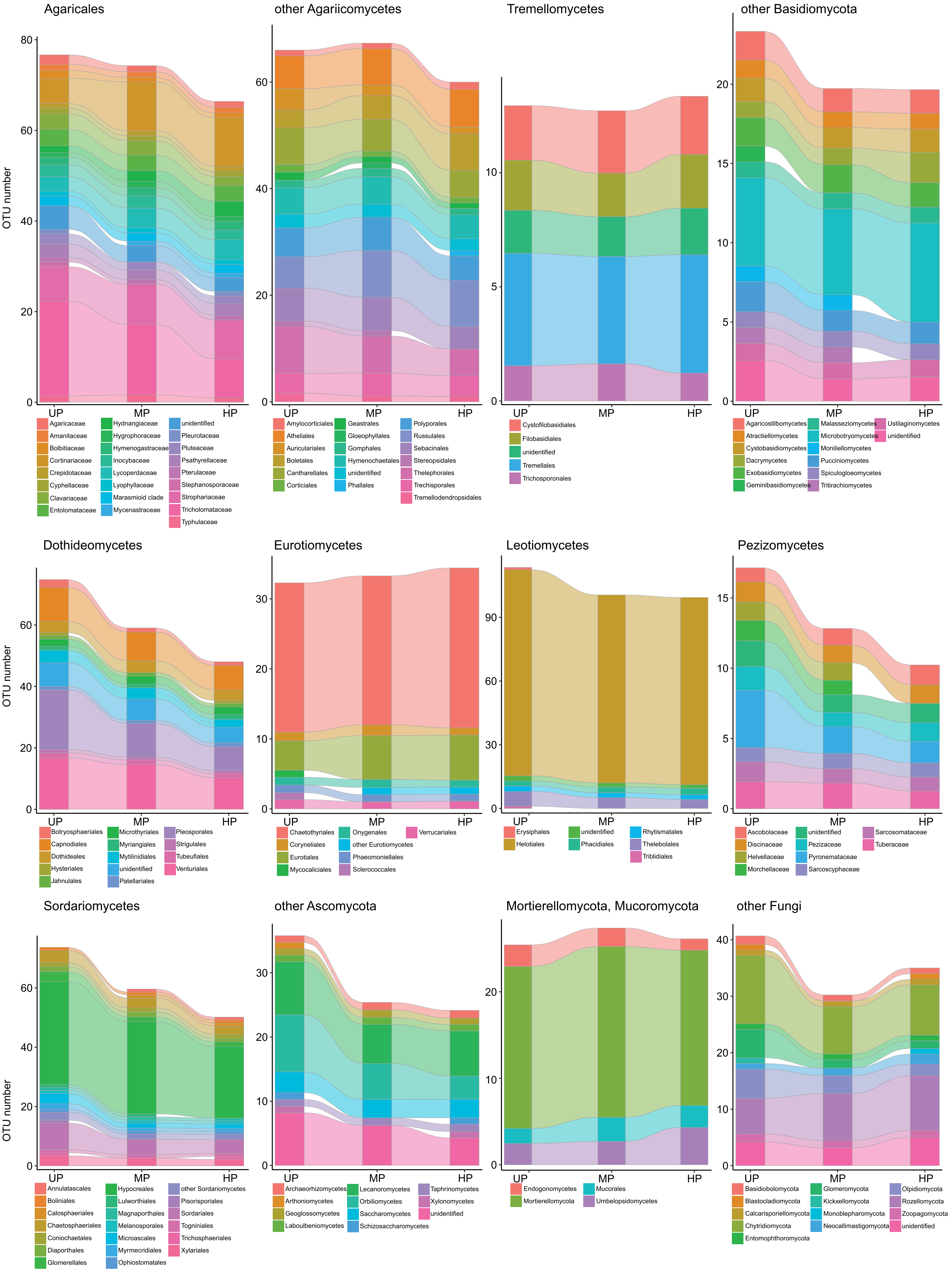

Figure S4. The average number of fungal OTUs in the litter collected close to CWD in unpolluted (UP), moderately polluted (MP), and heavily polluted (HP) areas (*n* = 29 samples per area).

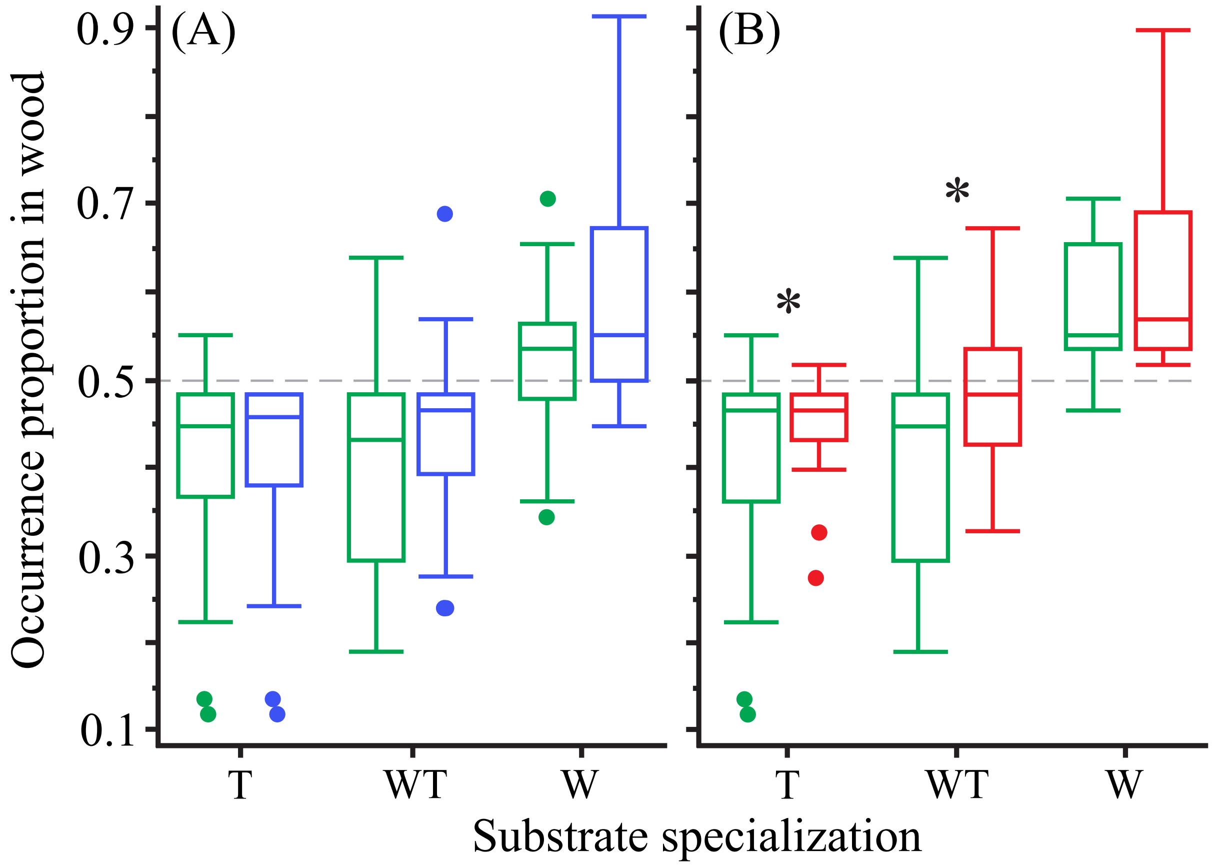

Figure S5. The proportion of occurrence of saprotrophic Tricholomataceae OTUs with different substrate specialization (T, terricolous; WT, wood-terricolous; W, wood-dwelling) in the wood in polluted areas. Comparisons of **(A)** OTU sets inhabiting UP (green) and MP (blue) areas and **(B)** OTU sets inhabiting UP (green) and HP (red) areas. Asterisks denote a significant difference in the fraction of OTUs occurrences in wood between two areas (GLMM, $p < 0.05$).

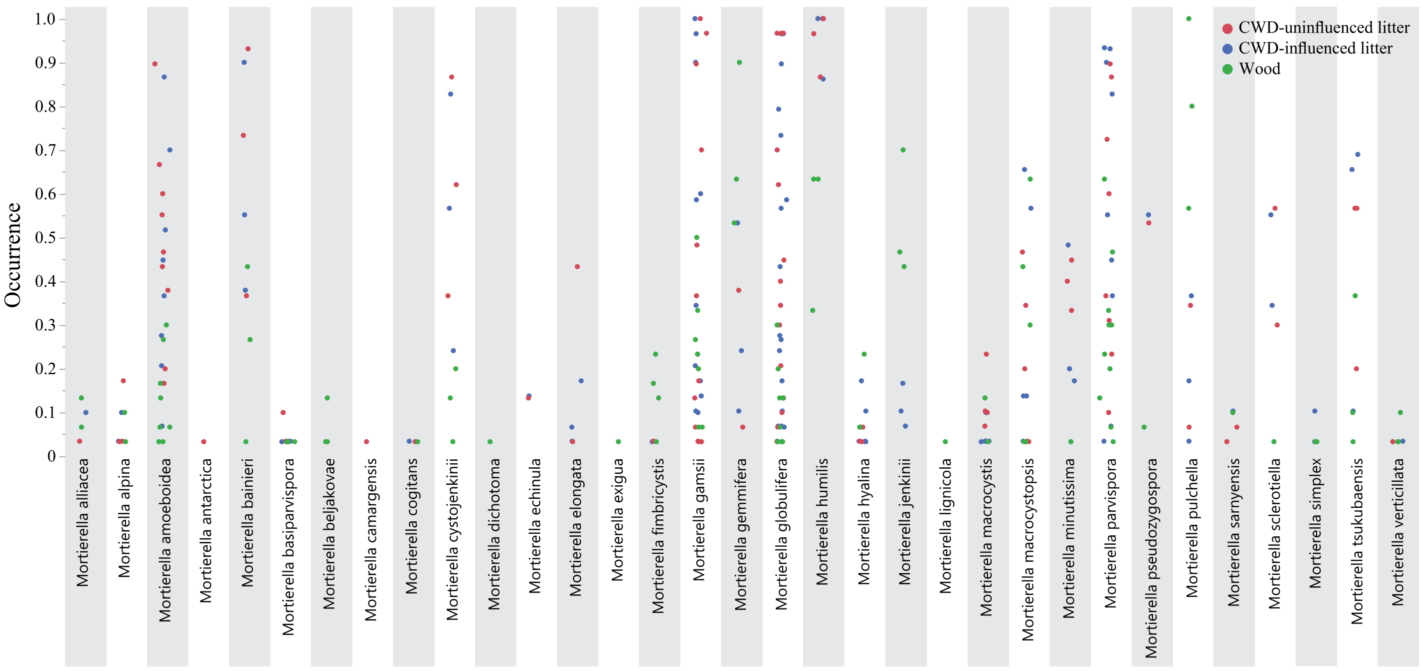

Figure S6. The occurrence of *Mortierella* species in wood, CWD-influenced, and CWD-uninfluenced forest litter.
